# GuREx-MIH: cost-effectiveness analysis of extraction versus restorative treatment for first permanent molars affected by Molar-Incisor Hypomineralisation in 11-year-old Swedish children

**DOI:** 10.1186/s12903-025-07633-4

**Published:** 2026-01-12

**Authors:** Adnan Hajdarević, Emina Čirgić, Birgitta Jälevik, Agneta Robertson, Tobias Fagrell, Mikael Svensson, Nina Sabel

**Affiliations:** 1 Department of Pediatric Dentistry, Institute of Odontology at the Sahlgrenska Academy, University of Gothenburg, Box 450, SE-405 30 Gothenburg, Sweden; 2 Folktandvården Björkekärr, Public Dental Service, Region Västra Götaland, Gothenburg, Sweden; 3 Institute of Odontology, Sahlgrenska Academy, University of Gothenburg, Gothenburg, Sweden; 4 Department of Orthodontics, Folktandvården Stockholms Län AB, Folktandvården Eastmaninstitutet, Stockholm, Sweden; 5 Department of Pediatric Dentistry, Institute of Odontology, Sahlgrenska Academy, University of Gothenburg, Gothenburg, Sweden; 6 Clinic of Pediatric Dentistry, University Dental Care, Public Dental Service, Region Västra Götaland, Gothenburg, Sweden; 7 School of Public Health and Community Medicine, Institute of Medicine, Sahlgrenska Academy, University of Gothenburg, Gothenburg, Sweden; 8 Department of Pharmaceutical Outcomes and Policy, College of Pharmacy, University of Florida, Gainesville, FL USA

**Keywords:** Developmental defects of enamel, Dental enamel hypomineralization, Pediatric dentistry, Health economics

## Abstract

**Background:**

The aim of this study was to evaluate the cost-effectiveness of extraction compared with restorative treatment of first permanent molars (FPMs) affected by Molar–Incisor Hypomineralisation (MIH) in 11-year-olds in Sweden.

**Methods:**

Seventy-five patients from the GuREx-MIH project were included. Of these, 43 were randomised to the Restorative treatment group (ReTG) and 32 to the Extraction treatment group (ExTG). Healthcare costs were calculated from dental records, while non-healthcare costs, were collected through surveys. Effectiveness was measured using the Child Perceptions Questionnaire (CPQ11-14) and the proportion of patients achieving a minimally important difference (MID). Incremental cost-effectiveness ratios (ICERs) were calculated as the difference in costs divided by the difference in effectiveness between groups.

**Results:**

The ExTG incurred higher mean healthcare (€1,417 vs. €1,051; *p* = 0.029) and total costs (€2,950 vs. €2,161; *p* = 0.029) compared with the ReTG, almost entirely due to general anaesthesia (GA), while non-healthcare costs were (€1,531 vs. €1,111; *p* = 0.117). When patients treated under GA were excluded, the ExTG became less costly, with lower healthcare costs (€868 vs. €1,051; *p* = 0.039), fewer visits (9.2 vs. 11.4; *p* = 0.025), and shorter chair time (324 vs. 401 min; *p* = 0.040). ICERs showed that with GA, extraction was associated with higher incremental costs per MID responder (€2,593 healthcare; €6,110 total). Without GA, extraction was either dominant when only healthcare costs were considered or showed a cost of €4,201 per MID responder when considering total costs.

**Conclusions:**

Compared with restorative treatment, extraction was associated with higher healthcare and total costs, primarily due to general anaesthesia, but also linked with a greater proportion of patients achieving clinically meaningful improvements in oral health-related quality of life at the age of 11 years.

**Trial registration:**

The study was retrospectively registered on ClinicalTrials.gov 29th of January 2024, registration number: NCT06228989.

**Supplementary Information:**

The online version contains supplementary material available at 10.1186/s12903-025-07633-4.

## Introduction

Molar-Incisor Hypomineralisation (MIH) affect the tooth enamel, and its aetiology remains unknown despite extensive research over the past two decades. It impacts between one and four first permanent molars (FPM) and can also involve the permanent incisors. Clinically, affected teeth display a clearly defined area of opacity, with more severe cases potentially experiencing post-eruptive breakdown [1]. Globally, around 14% of children are affected by MIH [2], with post-eruptive breakdown observed in 21% of the cases [3].

Affected teeth frequently present with hypersensitivity, particularly to cold foods and air inhalation, along with general dental discomfort [4]. Furthermore, MIH patients face an increased risk of developing caries, particularly in instances of post-eruptive breakdown [5]. The treatment requirement for affected FPMs varies based on patient symptoms and the severity of the defect [6]. Surveys among dentists indicate a lack of consensus regarding MIH treatment, including decisions on tooth extraction versus tooth preservation and the choice of restorative materials [7, 8]. As early as 1903, Angle discussed the importance of preserving the FPMs in dental literature, as they play a crucial role in establishing and maintaining the occlusion [9]. Although it represents the most invasive option, tooth extraction may offer the most favourable long-term prognosis, given that FPMs affected by MIH often require repeated restorative treatments, which frequently fail and may ultimately lead to extraction. [6].

The optimal age for FPM extraction is typically between 8 and 10 years, as this timing maximises the likelihood of spontaneous space closure and minimises the need for orthodontic intervention [10]. A model-based study by Elhennawy et al. (2017) showed that extracting severely affected MIH molars might be cost-effective if the timing of extraction is optimal, reducing the need for orthodontic therapy [11].

A previous study found no difference in self-reported oral health-related quality of life between patients who received restorative treatment and those who underwent extraction of first permanent molars affected by MIH [12]. Despite this, there is still considerable debate within the dental community regarding the optimal management of teeth with severe MIH. To date, there are no patient-based studies evaluating the cost-effectiveness of restorative treatment compared with extraction of such teeth. This highlights the need for prospective clinical studies exploring these treatment alternatives. This paper aims to assess the cost-effectiveness of extraction compared with restorative treatment of first permanent molars in patients with severe MIH at age 11 years.

## Material and methods

### Trial design and ethics

This cost-effectives analysis (CEA) was based on a trial aimed to monitor outcomes for patients with severe Molar-Incisor Hypomineralisation (MIH) in their first permanent molars (FPMs): The GuREx-MIH project (Gothenburg University Restoration or Extraction of First Permanent Molars with Severe MIH) [12]. The study was retrospectively registered on ClinicalTrials.gov: Clinical Trial Number: NCT06228989 with first posted date 29th of January 2024. The study was reported following the Consolidated Standards of Reporting Trials (CONSORT) and Consolidated Health Economic Evaluation Reporting Standards (CHEERS) guidelines [13, 14].

Participants and their guardians received detailed, age-appropriate information about the study. Informed consent was obtained from guardians after thorough verbal and written explanations. The study adhered to the ethical guidelines established by the World Medical Association’s Declaration of Helsinki and was approved by the Swedish Ethical Review Authority in Gothenburg (Dnr: 352–15).

### Participant recruitment

At baseline (T0), the sample consisted of 120 patients originating from a previous study, recruited from Public Dental Service in Region Västra Götaland and Region Östergötland, as well as from the Department of Pedodontics, Malmö University. Children aged 6–9 years were eligible if they had at least one FPM requiring treatment due to MIH, classified as grade 4 or 5. The MIH severity grading were based on the index described in detail by Hajdarević et al. (2025), which classifies MIH severity on a scale from degree 0 to 6. Degree 4 was defined as hypomineralised enamel with enamel breakdown or atypical restoration affecting ≤ 2 surfaces and associated symptoms, while degree 5 was defined as hypomineralised enamel with enamel breakdown or atypical restoration affecting more than 2 surfaces. Exclusion criteria at baseline included dental agenesis, chronic systemic illness, or functional impairments [12]. At 11-year follow-up (T1), additional exclusions were made if dental records were incomplete or missing.

### Treatment interventions

At T0, participants completed baseline assessments for oral health-related quality of life (OHRQoL). Treatment was allocated per randomisation and was performed using a computer-based random number generator [15]. Each participant was assigned a number between zero and nine; odd numbers were allocated to extraction and even numbers to restorative treatment with resin composite of the affected FPM. Sedation or general anaesthesia was administered when considered necessary, as determined by the attending dentist, as described elsewhere [12]. At T1, participants completed surveys for OHRQoL, and a health-economic survey. The latter was specifically designed to capture non-healthcare costs, including the number of caregiver attendances at dental visits, travel time to local clinics, and absences due to temporary parental leave to care for a sick child.

### Outcomes

#### Oral health-related quality of life

The primary dental health outcome was OHRQoL, assessed using the validated Swedish version of the short form of the CPQ11-14 [16].

OHRQoL was analysed in two ways. First, by comparing the difference in mean change scores between the treatment groups from baseline to follow-up. Second, by evaluating clinical relevance using the minimally important difference (MID). As no published MID value exists for the 16-item CPQ11-14 [17], it was estimated using a distribution-based approach, defined as half the standard deviation (0.5 × SD) of the change in scores between baseline and follow-up [17].

#### Cost measurements

In health economic evaluations, what is here defined as healthcare costs is commonly referred to as direct costs, while non-healthcare costs are often described as indirect costs. The combination of these, presented in this study as total costs, is traditionally termed the societal cost*,* reflecting the overall economic burden of a disease or treatment.

Healthcare costs were assessed using data extracted from patient records, which provided a detailed history of dental visits between the ages of 6 and 11 years, including the dates of appointments, treatments provided, and the duration of each visit. All estimates of chair time costs were calculated in Swedish currency (SEK) based on 2024 price levels: 2,375 SEK per hour for general dentists, and 775 SEK per hour for dental nurses, according to the Public Dental Service in Region Västra Götaland. The visits comprised follow-up examinations, supplementary assessments, treatment, re-treatment, and emergency visits, all carried out by general dentists, while dental acclimatisation and prophylactic appointments for FPMs were performed by dental nurses. The total cost per child was expressed in present value terms by annual discounting at a rate of 3% from T0 to T1.

The cost-effectiveness analysis excluded dental visits solely related to the study, interceptive orthodontic planning and treatment for malocclusions unrelated to FPM extraction, emergency visits and treatment for trauma, missed appointments, late cancellations, and treatment of other teeth.

The cost of general anaesthesia was calculated using a standard template based on the 2024 expenses of the Public Dental Service in Region Västra Götaland**.** This estimate covered the entire procedure, including preoperative preparation, anaesthesia induction, treatment time, and postoperative recovery. It also incorporated standardised hospital fees for anaesthesia services, which accounted for expenses related to anaesthesiologists, dental professionals, and nursing staff, material costs, facility use, postoperative monitoring, and administrative work. The total estimated standard cost was 16,215 SEK per general anaesthesia session.

Non-healthcare costs were evaluated using the health-economic survey, which included the number of caregiver attendances at dental visits, travel time to local clinics, and absences due to temporary parental leave to care for a sick child. In addition, a standard work absence on the day of treatment was assumed: 4 h for visits involving midazolam sedation and 8 h for those requiring general anaesthesia. The cost of lost productivity for caregivers was calculated using average standardised wage rates from Statistics Sweden (SCB) based on 2024 rates. The estimated cost of lost productivity, including social fees, was 328 SEK per hour of caregiver work absence [18].

Healthcare costs and non-healthcare costs were combined to calculate the total cost. Both healthcare costs and total costs were estimated with and without the inclusion of patients who underwent general anaesthesia. All costs were expressed in 2024 price levels and converted to Euros (€) using an exchange rate of 100 SEK = €8.74 [19].

### Cost-effectiveness analysis

A cost-effectiveness analysis was conducted to compare extraction with restorative treatment in terms of both costs and treatment outcomes. Treatment effect was assessed in two ways: first, by analysing the continuous change in OHRQoL using CPQ11-14 mean scores, and second, by calculating the proportion of patients who achieved a MID.

Cost-effectiveness was expressed as an incremental cost-effectiveness ratio (ICER), calculated as the difference in mean cost between the extraction treatment group (ExTG) and the restorative treatment group (ReTG) divided by the difference in treatment effect between the groups. ICER was reported in two ways: in Euros per CPQ11-14 point and in Euros per MID responder. Separate analyses of ICER were performed for total costs and for healthcare costs, as well as with and without the inclusion of patients treated under general anaesthesia.

$$ICER=\frac{(Cost\;of\;ExTG-Cost\;of\;ReTG)}{(Effect\;of\;ExTG-Effect\;of\;ReTG)}$$

### Statistical analysis

All statistical analyses were performed using IBM SPSS Statistics version 29.0 (IBM Corp., Armonk, NY, USA). Descriptive statistics were calculated for all baseline variables. Group differences were assessed using independent-samples t-tests for continuous variables and chi-squared tests for categorical variables. Changes in oral health-related quality of life (OHRQoL) were analysed using paired t-tests within groups and independent t-tests between groups.

Given that each patient could have between one and four affected teeth, multiple linear regression models were applied to examine the effect of treatment group (ExTG vs ReTG) on healthcare, non-healthcare, and total costs, adjusting for the number of treated teeth as a categorical variable. Regression coefficients were tested using two-sided t-tests, and subgroup analyses were performed excluding patients treated under general anaesthesia.

Uncertainty in ICERs and increments was quantified via non-parametric patient-level bootstrap with percentile 95% confidence intervals; when ExTG showed lower cost and greater effect (dominance), numeric ICERs were not presented by convention.

## Results

**Fig. 1:**
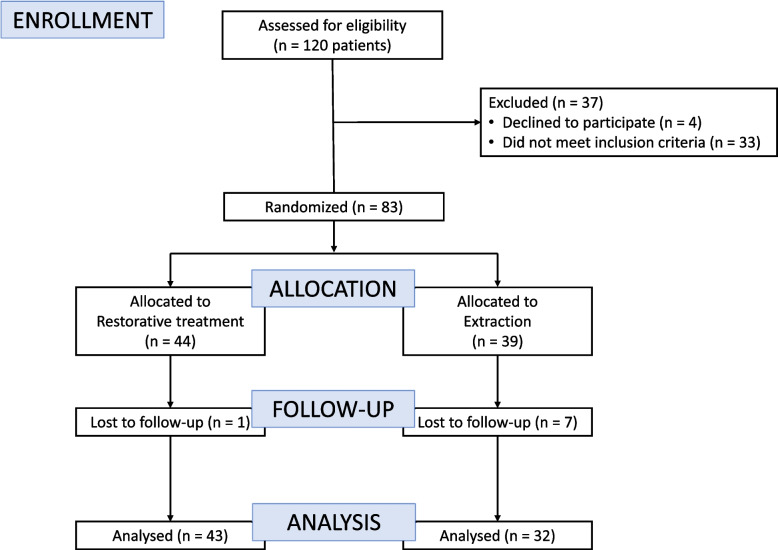
CONSORT Flow-chart of patients invited to participate

A total of 75 patients were included in the analysis, comprising 32 in the extraction treatment group (ExTG) and 43 in the restorative treatment group (ReTG). In the ExTG, 70 affected FPMs were treated, with a mean of 2.2 teeth per patient (SD = 1.0), while in the ReTG, 98 affected FPMs were treated, with a mean of 2.3 teeth per patient (SD = 1.0) (p = 0.686; t-test). (Fig. 1; Table 1). At baseline, the two treatment groups showed comparable number of decayed, extracted, and filled teeth in the primary dentition (deft) (Table 1). Re-treatment was required for 20 patients, during 33 sessions, in the ReTG.

**Table 1:** Demographic and clinical characteristics of patients in the restorative treatment (ReTG) and extraction treatment (ExTG) groups

Group	ReTG	ExTG	p-value
Patients, n	43	32	
Gender, n			
boys girls	20 23	12 20	0.586^1^
Treated FPMs (n)			
1 2 3 4	9 15 13 5	8 13 8 3	1.000^1^
deft at T0, mean (SD)	0.6 (1.5)	0.4 (1.5)	0.686^2^
CPQ11-14, mean (SD)			
T0 T1	9.3 (7.5) 8.6 (6.6)	12.1 (7.9) 9.3 (6.9)	0.136^2^ 0.684^2^

*FPM* first permanent molar, *n* number, *SD* standard deviation, *T0* baseline, *T1* follow-up.

^1^Chi-squared test ^2^T-test

Sedation with midazolam was used in 12 patients, during 16 sessions, in the ExTG and in 4 patients, during 6 sessions, in the ReTG. General anaesthesia was used in 11 patients, during 11 sessions, in the ExTG, while no patients in the ReTG received treatment under general anaesthesia.

### Oral health-related quality of life

The overall mean CPQ11-14 score decreased from 10.5 (SD = 7.7) at baseline (T0) to 8.9 (SD = 6.7) at follow-up (T1), corresponding to a mean change of −1.6 (SD = 6.5) for the total sample, indicating an improvement in oral health-related quality of life. The minimally important difference (MID) was 3.2 points.

When comparing the change in CPQ11-14 scores between groups, no significant differences were found. This remained the case after excluding patients treated under general anaesthesia. Similarly, no significant differences were observed between groups in the proportion of patients achieving a MID improvement, either before or after excluding those treated under general anaesthesia (Table 2).

**Table 2:** Comparison of, healthcare utilisation, costs, and treatment-related outcomes between the restorative treatment group (ReTG) and extraction treatment group (ExTG), presented both including all patients and excluding those treated under general anaesthesia

**Group**	**Including all patients**		**Excluding patients treated under general anaesthesia (GA)**	***p*-value ReTG vs. ExTG**	
	**ReTG mean (SD)**	**ExTG mean (SD)**	**ExTG mean (SD)**	**Including all patients**	**Excluding patients treated under GA**
Time spent at the dental office, min	401.1 (170.8)	346.7 (177.2)	324.2 (117.0)	0.187^2^	**0.040**^**2**^
Visits, n	11.4 (4.4)	9.4 (4.5)	9.2 (3.1)	0.058^2^	**0.025**^**2**^
Follow-up examination, n	3.5 (0.9)	3.4 (0.8)	3.5 (0.7)	0.614^2^	0.956^2^
Supplementary examination, n	0.3 (0.6)	0.5 (0.9)	0.4 (0.5)	0.381^2^	0.572^2^
Treatment, n	2.3 (1.0)	2.2 (0.9)	1.8 (0.7)	0.630^2^	**0.021**^**2**^
Re-treatment of FPM, n	0.8 (0.9)	0.0 (0.0)	0.0 (0.0)	**0.000**^**2**^	**0.000**^**2**^
Emergency visit due to FPM, n	0.4 (0.7)	0.3 (0.6)	0.3 (0.6)	0.507^2^	0.506^2^
Dental acclimatisation, n	1.0 (1.1)	1.2 (1.8)	1.3 (1.1)	0.570^2^	0.424^2^
Prophylactic visits of FPM, n	3.0 (2.7)	2.3 (2.9)	1.9 (2.3)	0.292^2^	0.106^2^
Parental work absence^1^, min	2,324.1 (1,627.9)	3,202.8 (2,774.7)	2,954.2 (2,915.1)	0.117^2^	0.364^2^
Healthcare cost, €	1,050.6 (424.7)	1,417.0 (845.2)	867.6 (260.2)	**0.029**^**2**^	**0.039**^**2**^
Non-healthcare cost, €	1,111.0 (778.6)	1,531.0 (1,326.6)	1,412.4 (1,393.7)	0.117^2^	0.364^2^
Total cost, €	2,160.7 (1,008.6)	2,950.1 (1,776.9)	2,278.3 (1,552.7)	**0.029**^**2**^	0.751^2^
Change in CPQ11-14	–0.7 (4.9)	−2.8 (8.1)	−1.8 (8.5)	0.207^2^	0.614^2^
MID responders, %	23%	38%	33%	0.279^3^	0.578^3^

^1^Including time spent on transportation to and from the clinic, time for dental treatment, and absences due to temporary parental leave to care for a sick child

^2^ T-test. ^3^Chi-square tests. Values in bold represent statistically significant association (*p* ≤ 0.05)

### Cost measurements

When comparing the ExTG with the ReTG, there were no differences in the total number of visits or in the total number of minutes spent in the dental chair. Healthcare costs were higher for patients in the ExTG, while non-healthcare costs were similar between groups. Consequently, the total cost was higher in the ExTG. After excluding patients who received treatment under general anaesthesia, the ExTG spent less time in the dental chair and had fewer total visits compared to the ReTG. Furthermore, the ExTG incurred lower healthcare costs. However, no difference was observed between the groups in terms of total cost (Table 2).

When including all patients, a higher number of treated teeth was associated with increased healthcare costs (*p* < 0.001) and total costs (*p* = 0.004), but not with non-healthcare costs (*p* = 0.760). After adjustment, patients in the ExTG had significantly higher healthcare costs than those in the ReTG (β = −4,758 SEK, *p* = 0.001), corresponding to mean group differences of 16,225 ± 9,672 SEK versus 12,012 ± 4,860 SEK. Similarly, total costs were higher in the ExTG (33,744 ± 20,320 SEK) compared to the ReTG (24,724 ± 11,535 SEK; p = 0.029; adjusted β ≈ −9,019 SEK, *p* = 0.007). For non-healthcare costs, no significant difference was observed between groups (17,519 ± 15,178 SEK vs. 12,713 ± 8,905 SEK; *p* = 0.117; adjusted p = 0.891). After excluding patients treated under general anaesthesia, the associations remained: a higher number of treated teeth was related to increased healthcare costs (*p* < 0.001) and total costs (*p* = 0.002), while no significant association was found for non-healthcare costs (*p* = 0.073). The treatment group itself was not associated with any of the cost outcomes.

### Cost-effectiveness analysis

In the cost-effectiveness analysis, the ExTG was consistently associated with higher costs compared to the ReTG, regardless of whether healthcare, non-healthcare, or total costs were considered. When using the change in CPQ11-14 scores as the effect outcome, extraction was linked to higher costs per additional improvement, and a similar trend was observed when MID improvement was used, with higher costs per additional responder in the ExTG group. These patterns were evident across all cost perspectives, and exclusion of patients treated under general anaesthesia did not alter the overall direction of results. Detailed ICER values with confidence intervals are presented in Table 3.

**Table 3:** Incremental cost-effectiveness ratios (ICERs) for extraction treatment group (ExTG) vs restorative treatment group (ReTG)

Population	Cost category	Effect outcome	ICER (€, point estimate)	95% CI (€, lower–upper)
Overall (incl. GA)	Healthcare	CPQ11–14 improvement	178	− 1,374 to 1,713
		MID responder	2585	− 14,646 to 26,184
	Non-healthcare	CPQ11–14 improvement	203	− 1,734 to 2,248
		MID responder	2949	− 15,946 to 29,863
	Total	CPQ11–14 improvement	381	− 3,083 to 4,009
		MID responder	5534	− 30,678 to 55,013
Excluding GA	Healthcare	CPQ11–14 improvement	186	− 1,362 to 1,346
		MID responder	1881	− 32,645 to 10,421
	Non-healthcare	CPQ11–14 improvement	296	− 2,796 to 2,720
		MID responder	2989	− 23,310 to 54,447
	Total	CPQ11–14 improvement	117	− 2,331 to 2,381
		MID responder	1181	− 26,289 to 32,799

### Drop-outs

Of the eight participants classified as lost to follow-up, seven actively withdrew from the study due to reluctance to attend the scheduled follow-up visit during the COVID-19 pandemic. One participant was excluded because essential dental records were unavailable, as the child had resided abroad for parts of the period between ages 6 and 11 years.

## Discussion

This study shows that both restorative and extraction treatments for first permanent molars (FPMs) affected by severe Molar-Incisor Hypomineralisation (MIH) improved oral health-related quality of life (OHRQoL) at the age of 11 years. In the primary analysis including all patients, extraction was associated with significantly higher healthcare and total costs, largely explained by the need for general anaesthesia, while differences in OHRQoL outcomes were not statistically significant. When patients treated under general anaesthesia were excluded in secondary analyses, extraction became more cost-efficient, suggesting that sedation requirements are a key determinant of the relative cost-effectiveness of treatment strategies. As the first trial to evaluate the cost-effectiveness of these treatment strategies, this study offers important insights for guiding clinical decision-making that considers both patient experiences and the efficient use of healthcare resources in the management of FPMs with MIH.

The observed improvement in CPQ11-14 scores in both groups is consistent with earlier findings showing that intervention, whether restorative or extraction, can improve symptoms related to MIH and enhance children's daily functioning and well-being [12, 20, 21]. Although the difference in OHRQoL change was not significant, the proportion of patients achieving a minimally important difference (MID) was higher in the extraction group. This suggests that extraction may lead to a more noticeable improvement in OHRQoL for some patients.

For the OHRQoL questionnaire, different approaches exist to define the MID, including anchor-based methods linked to patient-reported change and distribution-based methods using statistical variation. Anchor-based methods typically rely on patients rating whether their condition has improved, remained the same, or worsened. As this survey did not include such self-ratings, we applied a distribution-based approach, defining MID as half the standard deviation of the change scores, providing a pragmatic estimate in this context [17].

From a health economics perspective, the extraction treatment group initially showed higher total and healthcare costs, particularly when general anaesthesia was required. This is not unexpected, as surgical procedures are more resource-intensive. However, when patients treated under general anaesthesia were excluded, healthcare costs were lower in the extraction group, while patient-reported outcomes remained comparable to those of restorative treatment. Importantly, total costs did not differ substantially between groups, suggesting that extraction may represent a more resource-efficient option in many cases, if sedation requirements can be minimised. These findings align with model-based assumptions from Germany, which suggested that early extraction, if appropriately timed, can indeed be a cost-effective strategy [11].

The restorative treatment group required a considerable number of re-treatments, 33 teeth, reinforcing existing concerns about the limited durability of restorations in MIH-affected teeth [22, 23]. Previous research has reported median survival times of about five years for restorations in MIH-affected teeth [24]. Our findings are consistent with this and underline that even within a relatively short follow-up period, restorative care may lead to repeated interventions, adding not only to the clinical workload but also to the overall economic burden. This raises important questions about the long-term sustainability of restorative strategies in the management of severely MIH-affected molars. Higher caries experience is a well-documented risk factor for failure of restorative treatment [25, 26]. The patients in this study exhibited very low caries experience in the primary dentition, below the used threshold of deft ≤ 1 for low caries activity [27].

At the age of 11, it is difficult to determine whether orthodontic intervention will be necessary following the extraction of a FPM affected by MIH. However, research indicates a spontaneous space closure success rate of 84.3% in extraction cases, with a higher prevalence in the maxilla compared to the mandible [28]. Findings from a split-mouth trial showed that early extraction of compromised FPMs not only accelerates eruption but also good angulation of the maxillary second permanent molars, without leading to overeruption of antagonistic teeth by this age [29]. Together, these findings highlight that extraction, when timed appropriately, can promote favourable occlusal development and minimise the risk of unwanted orthodontic complications.

The use of sedation is a crucial consideration in the management of molars affected by MIH. In this trial, sedation added to overall costs but also facilitated treatment and reduced stress for children who might otherwise struggle with extensive procedures [30]. The choice of pharmacological support was based on a comprehensive clinical assessment, including dental fear and anxiety, treatment complexity, and previous dental experiences. Importantly, general anaesthesia was not routinely required for extraction of MIH-affected molars; the higher costs observed in the extraction group were primarily driven by its use in selected cases. In situations where extraction could be performed under local anaesthesia or minimal sedation, costs were substantially lower. It should also be noted that clinical practices regarding the use of sedation and general anaesthesia vary between countries, which may influence the generalisability of these cost findings [31]. From a health economics perspective, these additional costs are modest relative to total treatment expenses, yet they emphasise the need to balance financial implications against the clear benefits of improving the child’s treatment experience.

Non-healthcare costs related to caregiver productivity loss were similar across groups, indicating that differences in cost-effectiveness were driven primarily by the clinical procedures themselves rather than time away from work, and highlighting the importance of treatment modality, sedation requirements, and long-term maintenance. Given that the clinical effectiveness between restorative treatment and extraction was largely equivalent, and that overall costs were influenced mainly by the need for sedation, extraction may represent the more cost-effective option in selected cases. Importantly, it was not the extraction procedure itself that was costly, but rather the use of general anaesthesia, a finding confirmed by other studies [11, 32, 33]. Had general anaesthesia been required more frequently for restorative treatment, the associated costs of restorations would have increased correspondingly.

In the present trial, the comparison was focused on resin composite restorations and extraction, as these represent the most frequently chosen options for severely affected FPMs in Nordic clinical practice [7, 8]. Other treatments for MIH-affected FPMs, including ceramic restorations and stainless-steel crowns, have been shown to provide good long-term durability [23, 34].

This study also demonstrates the value of integrating real-world cost data and patient-reported outcomes in paediatric dentistry. The findings have implications not only for individual patient management but also for healthcare policy, particularly in publicly funded systems where justification of resource allocation is essential. Key strengths of the present study include the randomised design, prospective follow-up, and comprehensive cost calculations encompassing both healthcare and non-healthcare expenses. The use of validated instruments such as the CPQ11-14 further strengthens the measurement of treatment effects. Nonetheless, several limitations should be acknowledged. The sample size may have reduced statistical power, particularly for subgroup analyses. While malocclusions unrelated to FPM extraction were excluded, the children were too young at 11 years of age for a complete orthodontic assessment, and longer-term follow-up at older ages is needed to capture the clinical and economic consequences. In addition, it should be noted that restored FPMs may require further interventions over time, including repeated replacements of restorations, root canal treatments, prosthetic rehabilitation, or ultimately extraction at a later stage. A minor limitation was the loss of a small number of participants to follow-up. Another limitation concerns generalisability. This study was conducted within a Swedish public dental care system; therefore, cost structures and care pathways may differ in other countries.

Given the lack of clear long-term superiority of either treatment in terms of patient-reported outcomes, the choice between restorative and extraction treatment should be individualised, guided by tooth prognosis, patient preferences, and available resources. For patients unlikely to require general anaesthesia, early extraction, when appropriately timed, performed under local or minimal sedation, and feasible given the child’s ability to cooperate, may represent a resource-efficient alternative without compromising quality of life or clinical outcomes. Ultimately, treatment decisions should be made in close consultation with the child and their caregivers, taking into account psychological and developmental factors, as well as the likelihood of achieving successful long-term outcomes. These results support the integration of health economic perspectives into treatment planning for children with severe MIH.

## Conclusion

In this trial, both restorative and extraction treatment of severely MIH-affected first permanent molars improved oral health-related quality of life at the age of 11 years. Extraction was associated with higher healthcare and total costs, primarily due to the use of general anaesthesia, yet it also resulted in a greater proportion of patients achieving clinically meaningful improvements. Notably, when general anaesthesia was not required, extraction emerged as a resource-efficient and potentially more cost-effective option compared with restorative care.

## Supplementary Information

Supplementary Material 1.

Supplementary Material 2.

## Data Availability

The datasets used and analysed during the current study are available from the corresponding author on reasonable request.
